# Admission 25-Hydroxyvitamin D Deficiency Is Associated with Poor Outcomes in ICU Patients with Sepsis-Associated Encephalopathy (SAE)

**DOI:** 10.3390/jcm15145466

**Published:** 2026-07-13

**Authors:** Yajun Qian, Chen Qu, Hui Qi, Ying Xu

**Affiliations:** 1 Department of Intensive Care Unit, Nanjing Drum Tower Hospital, Affiliated Hospital of Medical School, Nanjing University, Nanjing 210008, China; qyj860410@163.com; 2 Geriatric Medicine Department, The Second Affiliated Hospital of Nanjing Medical University, Nanjing 210011, China; quchen@njmu.edu.cn

**Keywords:** 25-hydroxyvitamin D, sepsis-associated encephalopathy, mortality, interleukin-6

## Abstract

**Background**: Sepsis-associated encephalopathy (SAE) is a serious complication affecting septic patients and significantly increases mortality. Serum 25-hydroxyvitamin D (vitamin D) deficiency is prevalent in critical illness, but its relationship with SAE prognosis remains unclear. **Methods**: This retrospective cohort study enrolled SAE patients at Nanjing Drum Tower Hospital (January 2023–June 2024). Vitamin D levels were measured within 24 h of ICU admission with insufficiency and deficiency defined as ≤30 ng/mL and ≤20 ng/mL, respectively. Demographics, severity scores, laboratory parameters, organ support, and 90-day mortality were collected. Statistical analyses included Mann–Whitney U/*t*-tests, Spearman’s correlation, multivariate logistic regression, and ROC analysis. **Results**: Among 346 sepsis patients, 47 met SAE criteria. Vitamin D insufficiency affected 97.9% (46/47) of SAE patients, with 85.1% (40/47) being deficient. Ninety-day mortality was 25.5% (12/47). Non-survivors had significantly lower vitamin D levels than survivors (median 10.15 vs. 12.83 ng/mL, *p* = 0.010). Multivariate analysis identified higher APACHE II score (OR 1.164, 95% CI 1.042–1.300; *p* = 0.007) and lower vitamin D level (OR 0.758, 95% CI 0.603–0.954; *p* = 0.018) as independent predictors of 90-day mortality. Combining APACHE II score and vitamin D provided excellent predictive value (AUC 0.886). Vitamin D levels correlated negatively with IL-6 (rho = −0.293, *p* = 0.045) and vasopressor use (rho = −0.410, *p* = 0.004). **Conclusions**: Vitamin D insufficiency is nearly universal in SAE patients. Lower admission vitamin D levels independently predict 90-day mortality and enhance the predictive power of APACHE II. Vitamin D levels were negatively correlated with IL-6, suggesting a potential relationship with inflammation, but causality and clinical significance require further investigation.

## 1. Introduction

Sepsis-associated encephalopathy (SAE), a diffuse brain dysfunction secondary to systemic inflammation in sepsis, is characterized by acute cognitive impairment, delirium, or coma [1,2,3]. SAE is associated with increased mortality and long-term neurological sequelae [4]. Despite advances in sepsis management, the pathogenesis of SAE remains incompletely elucidated, and effective treatments are lacking [5,6].

The pathophysiology of SAE involves systemic inflammation triggering endothelial activation, disruption of the blood–brain barrier (BBB), neuroinflammation, and microglial activation. 25-hydroxyvitamin D (vitamin D) receptors are widely expressed on endothelial cells, astrocytes, and microglia. Experimental evidence indicates that vitamin D signaling can preserve BBB integrity by regulating tight junction proteins, reduce microglial activation, and attenuate the production of pro-inflammatory cytokines within the central nervous system. These properties suggest that vitamin D could be particularly relevant to SAE pathogenesis beyond its general immunomodulatory effects in sepsis.

Beyond its classical role in calcium and phosphate regulation, vitamin D is vital for immune function, endothelial health, and antimicrobial defense [7,8]. Vitamin D deficiency is highly prevalent among critically ill pediatric and adult patients [9,10] and is associated with impaired immunity, disrupted hormone metabolism, and increased susceptibility to severe infections, critical illness, and mortality [11,12,13,14,15]. Vitamin D supplementation increases serum vitamin D levels and may benefit critically ill patients, particularly those requiring mechanical ventilation (MV) [16]. Vitamin D also exerts neuroprotective effects, including facilitating the clearance of amyloid plaques, a hallmark of Alzheimer’s disease [17]. However, the relationship between vitamin D levels and SAE remains unclear. This study aimed to explore the association between vitamin D levels and prognosis in patients with SAE.

## 2. Materials and Methods

This retrospective study, approved by the institutional ethics committee with waived informed consent, enrolled patients diagnosed with SAE who were admitted to the intensive care unit (ICU) of Nanjing Drum Tower Hospital between January 2023 and June 2024.

### 2.1. Inclusion Criteria

(1) Meeting Sepsis-3 diagnostic criteria for sepsis; (2) diagnosed with SAE according to the criteria defined in Section 2.3; (3) age ≥ 18 years.

### 2.2. Exclusion Criteria

(1) Central nervous system infection; (2) brain trauma, cerebrovascular events, autoimmune encephalopathy, metabolic encephalopathy, or intoxication; (3) history of neurological deficits; (4) pregnancy; (5) missing clinical data.

### 2.3. Definition

Sepsis: diagnosed according to Sepsis-3 criteria.

SAE: Defined as either a Glasgow Coma Scale (GCS) score < 15 at admission in the absence of direct central nervous system infection, structural abnormalities, or other encephalopathies (e.g., hepatic, renal), or the emergence of delirium during hospitalization [1,2,3]. Diagnosis of delirium was based on the Confusion Assessment Method for the ICU (CAM-ICU). Assessments were conducted daily during the patient’s evaluable window of sedation status (Richmond Agitation-Sedation Scale score ≥ −3). A positive CAM-ICU result (indicating delirium) was defined as meeting all of the following criteria after excluding interference from deep sedation: acute change or fluctuation in mental status, inattention, and either disorganized thinking or altered level of consciousness.

GCS assessment was performed using the most reliable neurological evaluation available in the medical record. For patients not yet receiving sedation at the time of ICU admission, the GCS recorded immediately before sedative initiation was used as the baseline. For patients who were already intubated and receiving continuous sedation, the GCS documented during daily sedation interruption (DSI) assessments was utilized, as DSI is routinely performed in our ICU unless contraindicated. If sedation could not be safely interrupted, these patients were excluded from the primary GCS-based SAE definition, as persistent deep sedation confounds mental status evaluation. However, they remained eligible for inclusion if they subsequently developed delirium (assessed during sedation-free windows) during their ICU stay.

To rule out alternative causes of encephalopathy, we systematically excluded the following conditions based on clinical assessment and available laboratory/imaging data: hepatic encephalopathy (normal ammonia or no acute liver failure), severe uremia (azotemia not primary), electrolyte disturbances (sodium, calcium, magnesium with no clinically significant abnormalities), other metabolic causes (glucose, thyroid function, and thiamine levels with no clinically significant abnormalities), structural brain lesions (neurological examination and neuroimaging), and central nervous system infection (no meningeal signs, negative cerebrospinal fluid studies when performed, or no clinical suspicion). Only patients with no identifiable alternative etiology were classified as SAE.

Vitamin D Status: insufficiency and deficiency were defined as serum vitamin D ≤ 30 ng/mL and ≤20 ng/mL, respectively, based on published literature [18,19].

### 2.4. Data Collection

Within 24 h of admission, the following data were recorded: demographic characteristics (gender, age); severity scores including the Acute Physiology and Chronic Health Evaluation (APACHE) II and Sequential Organ Failure Assessment (SOFA) as well as the GCS scores, laboratory parameters comprising white blood cell count (WBC), neutrophil percentage (N%), lymphocyte percentage (LY%), interleukin-6 (IL-6), heparin-binding protein (HBP), procalcitonin (PCT), human leukocyte antigen-DR (HLA-DR), cluster of differentiation 4 (CD4), cluster of differentiation 8 (CD8), alanine aminotransferase (ALT), total bilirubin (TB), blood urea nitrogen (BUN), serum creatinine (SCr), ferritin, vitamin A, vitamin B1, vitamin E, vitamin K1 and vitamin D; and utilization of life-sustaining treatments such as MV, continuous renal replacement therapy (CRRT), extracorporeal membrane oxygenation (ECMO) and vasoactive drugs.

### 2.5. Clinical Endpoints: 28-Day and 90-Day Mortality

Ninety-day mortality was selected as the primary endpoint for the following reasons: (i) it is a robust long-term outcome measure in critical care research, capturing both in-hospital and post-discharge deaths attributable to sepsis; (ii) major randomized trials in sepsis and vitamin D supplementation have used 90-day mortality as a primary or key secondary endpoint, facilitating comparison with our findings; (iii) 28-day mortality may underestimate sepsis-related deaths, as patients can survive the acute phase but die later from persistent organ dysfunction or secondary infections; and (iv) late deaths occurring between day 28 and day 90 are not uncommon in septic patients. For completeness, we also report 28-day mortality as a secondary endpoint.

### 2.6. Statistical Methods

Complete-case analysis was used, as missing data were minimal (<5% for all variables) and unlikely to introduce bias. Statistical analyses were performed using SPSS 26.0. The Shapiro–Wilk test was used to assess the normality of continuous variables; variables with *p* > 0.05 were considered normally distributed. Normally distributed data are presented as mean ± standard deviation (SD) and were compared using independent samples *t*-tests. Non-normally distributed data are expressed as median (interquartile range) [M (P25, P75)] and were compared using the Mann–Whitney U test. Categorical variables are described as frequency (percentage) [n (%)] and were compared using the chi-square test.

Associations between variables were assessed using Spearman’s rank correlation. Variables with *p* < 0.05 in univariate analysis were considered candidates for the multivariable model. Given the limited number of mortality events (n = 12), we restricted the final model to a maximum of two predictors to maintain an events-per-variable ratio ≥ 5:1; vitamin D was forced into the model based on an a priori hypothesis, and the additional predictor was selected from the remaining candidates. Multivariate logistic regression analysis was employed to identify independent risk factors for 90-day mortality.

The diagnostic performance of biomarkers was evaluated using receiver operating characteristic (ROC) curve analysis to determine sensitivity, specificity, and optimal cut-off values. Because vitamin D levels were inversely associated with mortality, ‘90-day survival’ was defined as the positive state in the ROC analysis to ensure clinically meaningful interpretation. To assess the stability of the regression coefficients and potential overfitting, we performed bootstrap internal validation with 1000 resamples, using bias-corrected and accelerated (BCa) 95% confidence intervals.

A two-sided *p*-value < 0.05 was considered statistically significant.

## 3. Results

### 3.1. Clinical Characteristics of Patients

Of the 346 patients with sepsis, 47 (13.6%) were diagnosed with SAE, including 31 males and 16 females. The primary sources of infection included pneumonia (n = 22, 46.8%), bacteremia (n = 12, 25.5%), intra-abdominal infection (n = 10, 21.2%), urinary tract infection (n = 4, 8.5%), biliary tract infection (n = 3, 6.3%), and skin/soft tissue infection (n = 2, 4.2%). Because some patients had concurrent infections at multiple sites, the sum of the percentages for individual infection sources exceeds 100%. During the ICU course, 23 patients (48.9%) received mechanical ventilation, 17 (36.1%) underwent continuous renal replacement therapy, 21 (44.6%) required vasoactive agents, and 2 (4.2%) were supported with extracorporeal membrane oxygenation; epileptic seizures occurred in 3 patients (6.3%). The 28-day and 90-day mortality rates were 17.0% (n = 8) and 25.5% (n = 12), respectively.

Regarding baseline characteristics, the median Charlson Comorbidity Index was 4 (IQR 1–6). Chronic kidney disease (eGFR < 60 mL/min/1.73 m^2^ for >3 months) was present in five patients (10.6%), and chronic liver disease (mostly cirrhosis, Child–Pugh class A or B) in five patients (10.6%). All patients (n = 47) had an NRS-2002 score ≥ 3, as every patient had an APACHE II score ≥ 10, which automatically confers a disease-severity score of 3 on the NRS-2002; thus, the prevalence of nutritional risk in this cohort was 100%. Vitamin D insufficiency (≤30 ng/mL) was observed in 46 patients (97.9%), and deficiency (≤20 ng/mL) in 40 patients (85.1%); only one patient reported taking vitamin D supplements prior to admission.

### 3.2. Association Between Vitamin D Status and Organ Support Requirements in SAE Patients

We then assessed whether vitamin D deficiency (≤20 ng/mL vs. >20 ng/mL) was associated with the need for organ support. No significant differences were observed between the two groups in the requirement for MV, CRRT, vasoactive agents, or ECMO (Table 1).

### 3.3. Comparison of Demographic and Laboratory Characteristics Between Survivors and Non-Survivors

When stratified by 90-day survival status, no significant differences were observed between survivors and non-survivors in terms of age, sex, or laboratory parameters, including WBC, N%, LY%, IL-6, ferritin, HBP, PCT, HLA-DR, CD4/CD8 ratio, ALT, TB, BUN, and SCr. However, non-survivors had significantly higher APACHE II and SOFA scores, as well as lower GCS scores. Vitamin E and vitamin D levels were significantly lower in non-survivors, whereas levels of vitamins A, B1, and K1 did not differ significantly between the two groups (Table 2).

### 3.4. Independent Predictors of 90-Day Mortality in SAE Patients

A total of 23 variables were entered into univariate analysis (Table 2 and the accompanying text). These included demographic characteristics (age, sex), severity scores (APACHE II, SOFA, GCS), laboratory parameters (WBC, N%, LY%, IL-6, ferritin, HBP, PCT, HLA-DR, CD4/CD8 ratio, ALT, TB, BUN, SCr), and vitamin levels (vitamins A, B1, D, E, K1).

Univariate analysis identified six candidate variables with *p* < 0.05: APACHE II score, SOFA score, GCS, TB, vitamin E, and vitamin D. APACHE II score and admission vitamin D level were subsequently entered into the final multivariable logistic regression model. Multivariate analysis revealed that both APACHE II score and admission vitamin D level were independent predictors of 90-day mortality (Table 3).

### 3.5. Predictive Value of Vitamin D for 90-Day Mortality

**Figure 1:**
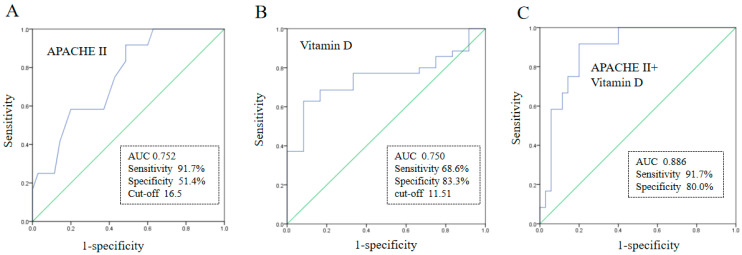
Receiver operating characteristic (ROC) curves for predicting 90-day mortality in SAE patients. ROC curves showing the predictive value of different factors for 90-day mortality in SAE patients. (**A**) APACHE II score; (**B**) Vitamin D level; (**C**) APACHE II score combined with vitamin D level.

ROC analysis showed that the AUC for APACHE II in predicting 90-day mortality was 0.752 (95% CI: 0.604–0.900; *p* = 0.01), and for vitamin D was 0.750 (95% CI: 0.610–0.890; *p* = 0.01). The optimal cut-off values were 16.5 for APACHE II (sensitivity 91.7%, specificity 51.4%) and 11.51 ng/mL for vitamin D. In the ROC analysis, ‘90-day survival’ was defined as the positive state; the sensitivity for predicting survival was 68.6% (24/35), and the specificity for predicting mortality was 83.3% (10/12) (Figure 1A,B).

Combining APACHE II score and vitamin D level yielded an AUC of 0.886 (95% CI 0.789–0.982) (Figure 1C), demonstrating enhanced predictive utility for 90-day mortality in SAE patients. Formal comparison using DeLong’s test confirmed that the combined model achieved significantly better discrimination than the APACHE II-alone model (AUC 0.886 vs. 0.752, *p* = 0.025).

To evaluate the stability of the multivariable logistic regression model, we performed bootstrap resampling (1000 samples) with bias-corrected and accelerated (BCa) confidence intervals. The original coefficient for vitamin D was −0.277. The bootstrap mean coefficient was −0.277, and the BCa 95% confidence interval was −0.615 to −0.150, which did not cross zero. The BCa 95% CI for APACHE II was 0.053–0.413, also excluding zero. These results confirm that both predictors remained statistically significant and directionally consistent across repeated resampling, supporting the robustness of the model despite the limited sample size.

### 3.6. Relationship Between Vitamin D Levels and Clinical Indicators

Vitamin D levels were significantly negatively correlated with IL-6 levels (rho = −0.293, *p* = 0.045) and vasopressor use (rho = −0.410, *p* = 0.004). A negative correlation was also observed with 90-day mortality (rho = −0.378, *p* = 0.009). No significant correlations were found between vitamin D levels and other clinical indicators.

Furthermore, to determine whether the prognostic value of vitamin D was independent of general illness severity, we assessed its correlation with critical care scores. No significant correlations were observed between serum vitamin D levels and either the APACHE II score (rho = 0.016, *p* = 0.914) or the SOFA score (rho = −0.063, *p* = 0.674).

## 4. Discussion

Sepsis-associated encephalopathy (SAE) is a major manifestation of organ dysfunction in sepsis, with reported incidences ranging from 9% to 71% in severe sepsis [20]. In our cohort of 346 sepsis patients, the SAE incidence was 13.6% (47/346), consistent with prior reports [21]. Using the MIMIC-III database, Ruan et al. reported a 28-day mortality of 11% [22], while our study observed 28-day and 90-day mortality of 17.0% and 25.5%, respectively. Established risk factors for SAE mortality include age, chronic comorbidities, non-neurological SOFA score, and severe GCS impairment [23]. Consistently, our non-survivors had significantly higher APACHE II and SOFA scores and lower GCS scores, confirming illness severity as a critical determinant.

For multivariable analysis, we selected APACHE II over SOFA because it has been more extensively validated for mortality prediction, specifically in SAE, and better captures the prognostic impact of neurological impairment (via GCS) and chronic comorbidities. Although GCS and total bilirubin were significant in univariate analysis, they were not included as separate covariates because they are integral components of APACHE II. Vitamin E was also excluded despite its univariate association (*p* = 0.010), as all values were within normal range, no consistent literature links it to SAE mortality, and adding it would have reduced the events-per-variable ratio from 6:1 to 4:1. By contrast, vitamin D was forced into the model based on our a priori hypothesis, given its established roles in neuroinflammation and blood–brain barrier integrity.

After adjusting for APACHE II, vitamin D remained independently associated with 90-day mortality. Importantly, we found no significant collinearity between vitamin D levels and APACHE II or SOFA scores. This observation supports the notion that the association between low vitamin D and mortality is not merely a reflection of greater acute illness severity. However, this finding should be interpreted with caution. Vitamin D levels in critically ill patients are influenced by multiple confounders—fluid resuscitation, hepatic and renal dysfunction, CRRT, and prolonged ICU stay with limited sunlight exposure [24,25]. Furthermore, the measured total 25(OH)D levels are strongly influenced by vitamin D-binding protein and albumin concentrations, both of which are frequently altered in critical illness; thus, total vitamin D levels may not accurately reflect the biologically available fraction in this population.

Among other vitamins, vitamin B1 (thiamine) showed no significant difference between survivors and non-survivors in our cohort. This aligns with previous reports: Corcoran et al. found no correlation between thiamine levels and inflammation or mortality [26], and Sedhai et al. reported that thiamine supplementation reduced ICU delirium but conferred no mortality benefit [27].

The pathophysiology of SAE involves neuroinflammation, blood–brain barrier disruption, oxidative stress, and mitochondrial dysfunction. Vitamin D has been shown to inhibit pro-inflammatory cytokines (IL-2, IL-6, IL-8, TNF-α) and modulate microglial activation [28,29]. In our study, vitamin D levels were negatively correlated with IL-6 (rho = −0.293, *p* = 0.045). Nevertheless, given that IL-6 levels did not differ between survivors and non-survivors, the observed correlation alone cannot support a causal IL-6-mediated effect on mortality. Immunomodulatory explanations remain hypothesis-generating and warrant further investigation.

To contextualize the strikingly high prevalence of vitamin D deficiency in our SAE cohort (insufficiency 97.9%, deficiency 85.1%), we compared it with general population data. A national multicenter study reported a mean serum 25(OH)D level of 26.00 ng/mL in Chinese adults, with deficiency (<20 ng/mL) affecting 29.89% [30]. In the Nanjing region, a study of 3326 residents reported a deficiency rate of 63.3% [31]. A recent meta-analysis reported that 55–76% of sepsis patients have vitamin D deficiency or insufficiency [32]. Our SAE cohort exhibited substantially higher rates, suggesting that SAE patients represent an extremely high-risk subgroup, likely due to critical illness–related metabolic changes, reduced sunlight exposure, and the underlying disease process itself.

The near-universal prevalence of deficiency in our cohort raises important considerations for clinical utility and statistical inference. When analyzed as a continuous variable, vitamin D remained significantly associated with mortality, suggesting that within the deficient range, lower levels still carry prognostic information. However, the restricted range of values (most between 8 and 20 ng/mL) may attenuate correlation coefficients—a “spectrum restriction” that could explain the lack of significant correlations with several organ support measures. External validation in cohorts with broader vitamin D ranges is needed. The optimal cutoff identified in our ROC analysis (11.51 ng/mL) should not be interpreted as a clinically actionable decision threshold in this near-universally deficient cohort; its clinical utility requires prospective validation in independent populations before any threshold-based application can be considered.

It is also critical to emphasize that our observational study cannot establish causality. Large RCTs in critically ill patients have shown no mortality benefit from high-dose vitamin D supplementation: the VITdAL-ICU trial found no significant reduction in hospital length of stay or mortality [33], and the VIOLET trial demonstrated no advantage over placebo for 90-day mortality [34]. This creates an important context: low baseline vitamin D is a strong prognostic marker in the high-risk SAE subgroup, but universal, non-selective “rescue” therapy initiated after critical illness onset offers limited survival benefit. Our findings should therefore be interpreted as identifying vitamin D as a prognostic marker—a surrogate for illness severity, chronic nutritional status, or systemic inflammation—rather than as evidence for a direct therapeutic target. The lack of correlation with severity scores should not be overinterpreted as evidence of a distinct pathophysiological role, as residual confounding remains possible. Future interventional research should investigate early correction of deficiency in high-risk patients or even preventive maintenance of adequate levels.

As the first study to investigate the relationship between vitamin D levels and SAE prognosis, this research has several limitations. First, its single-center, retrospective design limits generalizability, and the relatively small SAE cohort (n = 47, with only 12 deaths) raises concerns about statistical overfitting. Although bootstrap internal validation supported model stability (both predictors had BCa 95% CIs excluding the null), the limited events preclude definitive conclusions. Second, the near-universal prevalence of deficiency restricted variability and statistical power. Third, we could not perform external validation due to lack of an independent SAE cohort. Fourth, residual confounding cannot be excluded despite adjustment for APACHE II. Fifth, we acknowledge that our variable selection may have introduced bias; future larger studies should explore the independent role of vitamin D in SAE prognosis. Sixth, serum 25(OH)D levels were measured using a chemiluminescence immunoassay (CLIA), which is subject to inter-assay and inter-laboratory variability. This may affect the precision of the absolute cut-off (11.51 ng/mL) identified by our ROC analysis. Despite these limitations, our findings provide the first evidence that vitamin D is independently associated with 90-day mortality in SAE patients, and lay the groundwork for future prospective, multicenter studies to validate vitamin D as a prognostic biomarker and to explore whether early correction of deficiency improves outcomes in this high-risk population.

## 5. Conclusions

Vitamin D insufficiency is highly prevalent among SAE patients. Lower admission vitamin D levels and higher APACHE II scores are independent predictors of 90-day mortality, and their combination provides a strong predictive model. Vitamin D levels were negatively correlated with IL-6, suggesting a potential relationship with inflammation. However, given the observational design and the results of large randomized trials that failed to demonstrate a survival benefit from vitamin D supplementation in critical illness, vitamin D status should be interpreted primarily as a prognostic marker rather than as evidence of a direct causal pathway or a rationale for immediate therapeutic supplementation. Causality and independence from unmeasured confounders cannot be established from this observational study. Our findings are hypothesis-generating and require validation in prospective studies and, if supported, interventional trials specifically targeting SAE patients.

## Figures and Tables

**Table 1:** Organ Support Requirements by Vitamin D Status in SAE patients.

Variable	Vitamin D ≤ 20 ng/mL (n = 40)	Vitamin D > 20 ng/mL (n = 7)	OR	95% CI	*p*
Ventilation, n (%)	19 (47.5)	4 (57.1)	0.68	0.13–3.43	0.951
CRRT, n (%)	14 (35.0)	3 (42.8)	0.72	0.14–3.76	0.978
Vasopressor, n (%)	20 (50.0)	1 (14.2)	6.00	0.66–54.43	0.180
ECMO, n (%)	2 (5.0)	0 (0)	0.97	0.04–22.44	0.682

**Table 2:** Characteristics of SAE Survivors and Non-Survivors (90-Day).

Variable	Survivors (n = 35)	Non-Survivors (n = 12)	*p*
Sex, Male, n (%)	23 (65.70)	8 (66.70)	0.770
Age, years	61.00 ± 19.00	59.10 ± 24.30	0.783
APACHE II	16.00 (12.00, 24.00)	25.50 (18.50, 31.80)	0.010
SOFA	7.00 (3.00, 10.00)	10.50 (9.00, 14.50)	0.013
GCS	14.00 (10.00, 14.00)	12.00 (5.30, 13.00)	0.029
WBC, ×10^9^/L	10.06 ± 4.43	12.57 ± 7.53	0.294
N%	82.70 (78.10, 87.10)	86.75 (79.33, 92.43)	0.257
LY%	9.50 (5.90, 13.60)	7.95 (5.35, 14.23)	0.626
IL-6, pg/mL	78.40 (35.93, 166.70)	42.54 (11.63, 105.20)	0.172
Ferritin, μg/L	366.00 (173.50, 532.00)	1262.50 (120.50, 1630.50)	0.112
HBP, ng/mL	45.86 (22.90, 88.75)	27.00 (10.63, 104.80)	0.425
PCT, ng/mL	0.68 (0.10, 2.67)	1.27 (0.52, 2.42)	0.464
HLA-DR, %	42.90 (23.80, 54.40)	30.25 (14.68, 47.93)	0.192
CD4/CD8	1.34 (0.93, 1.87)	1.18 (0.71, 2.68)	0.779
ALT, U/L	19.70 (12.00, 43.00)	45.40 (19.18, 114.00)	0.088
TB, μmol/L	12.50 (7.80, 21.70)	19.85 (13.18, 204.75)	0.040
BUN, mmol/L	8.10 (6.20, 12.30)	9.90 (6.83, 21.50)	0.262
SCr, μmol/L	81.00 (59.00, 213.00)	143.50 (44.25, 238.25)	0.961
Vitamin A, μg/L	246.67(119.77, 366.02)	157.29 (128.01, 198.38)	0.817
Vitamin B1, μg/L	0.92 (0.61, 1.69)	0.64 (0.50, 1.20)	0.156
Vitamin E, μg/mL	6.83 (6.19, 8.74)	5.77 (4.32, 6.48)	0.010
Vitamin K1, μg/L	0.46 (0.20, 0.90)	0.48 (0.26, 1.44)	0.420
Vitamin D, ng/mL	12.83 (10.60, 18.54)	10.15 (8.55, 11.36)	0.010

Data presented as Mean ± SD, Median (Q1, Q3), or n (%).

**Table 3:** Multivariate Analysis of Risk Factors for 90-Day Mortality in SAE Patients.

Variables	β	S.E	Wald	*p*-Value	OR	95%CI	
						Lower	Upper
APACHE II	0.152	0.056	7.225	0.007	1.164	1.042	1.300
Vitamin D	−0.277	0.117	5.564	0.018	0.758	0.603	0.954

## Data Availability

The data are available from the corresponding author upon reasonable request.

## Abbreviations

SAE	sepsis-associated encephalopathy
Vitamin D	25-hydroxyvitamin D
MV	mechanical ventilation
ICU	intensive care unit
GCS	Glasgow Coma Scale
APACHE II	Acute Physiology and Chronic Health Evaluation II
SOFA	Sequential Organ Failure Assessment
WBC	white blood cell count
N%	neutrophil percentage
LY%	lymphocyte percentage
IL-6	interleukin-6
HBP	heparin-binding protein
PCT	Procalcitonin
HLA-DR	human leukocyte antigen-DR
CD4	cluster of differentiation 4
CD8	cluster of differentiation 8
ALT	alanine aminotransferase
TB	total bilirubin
BUN	blood urea nitrogen
SCr	serum creatinine
CRRT	continuous renal replacement therapy
ECMO	extracorporeal membrane oxygenation
SD	standard deviation
ROC	receiver operating characteristic
